# Use of gonadotropin-releasing hormone (GnRH) agonist trigger in fertility preservation for patients with inherited genetic disorders

**DOI:** 10.3389/fendo.2022.826419

**Published:** 2022-09-06

**Authors:** Jamie Merkison, Carrie Malcom, Alan Decherney

**Affiliations:** ^1^ Eunice Kennedy Shriver National Institute of Child Health and Human Development, The National Institutes of Health, Bethesda, MD, United States; ^2^ Department of Obstetrics and Gynecology, Jackson Health System, Miami, FL, United States

**Keywords:** GnRH, fertility preservation, GnRH agonist, oocyte cryopreservation, OHSS

## Abstract

In patients with varying hematologic disorders (thalassemia, sickle cell anemia, aplastic anemia, etc.), inherited bone marrow failure syndromes, and immune deficiencies due to a single gene disorder, the advent of stem cell transplantation (SCT) as a treatment option has allowed for significant disease improvement, and possibly cure. This specific treatment option often requires exposure to chemotherapeutic agents and sometimes whole body radiation; therefore, primary ovarian insufficiency is often sequelae of the therapy. The optimization of fertility preservation protocols within this patient population is of extreme importance. This review aims to detail the use of GnRH agonist use within this patient population, within the context of fertility preservation cycles.

## Introduction

For patients with hematologic malignancies and hematologic benign diseases such as thalassemia, aplastic and sickle cell anemia, inherited bone marrow failure syndromes and immune deficiency disorders, hematopoietic stem cell transplantation (HSCT) can lead to disease improvement and possibly cure (1, 2). However, chemotherapeutic agents and whole-body irradiation given during the “conditioning” phase just prior to stem cell transplantation are often toxic to the ovaries, leading to premature ovarian insufficiency and loss of fertility, in an estimated >80% of patients (1, 3, 4). Fertility preservation is often a concern for these patients, especially considering approximately 45% of allogeneic HSCT recipients and 25% autologous HSCT patients are younger than 40 years old when they receive the transplant (5).

In pre-pubertal females, ovarian tissue cryopreservation is currently the only option for fertility preservation. In post-pubertal girls, oocyte cryopreservation is an additional option prior to HSCT. Prospective studies in these patients have shown that a small portion of patients may retain their fertility following HSCT, however the risk of premature ovarian sufficiency remains high, and it is difficult to predict who will retain the fertility and for how long, though choice of conditioning chemotherapy regimen plays a role (3, 4). Likewise, patients with solid tumors are at an increased risk of premature ovarian sufficiency and infertility following chemotherapy administration. These patients are also candidates for fertility preservation prior to treatment initiation. The introduction of the GnRH-agonist trigger has become an important aspect of maintaining safety within modern protocols, especially within the context of fertility preservation.

## Physiology of ovulation and assistive reproductive technologies

During the menstrual cycle, gonadotropin-releasing hormone (GnRH) is released from the hypothalamus in both pulsatile and surge fashions due to the pulsatile nature of the GnRH releasing neuron (6). The primary site of action of GnRH is the pituitary gland, causing secretion of follicular stimulating hormone (FSH) and luteinizing hormone (LH). The main function of FSH is in survival of maturing ovarian follicles (6). FSH stimulates follicular growth and estrogen secretion in the ovaries which causes cumulus cell expansion around the oocytes and release of proteolytic enzymes, allowing the oocyte to separate from the follicular wall (7, 8). LH also plays a role in follicle maturation. As estrogen levels increase, at mid cycle there is a surge in LH and FSH from the pituitary gland, triggering ovulation approximately 36 hours later (7). LH then triggers progesterone production from the corpus luteum, which is vital for endometrial maturation and facilitation of embryo implantation (6).

Assistive reproductive technologies (ART) mimic this physiological process to induce ovulation.

Several stimulation protocols incorporate the use of GnRH agonists beginning in the mid-luteal phase to serve as pre-stimulation downregulation, commonly referred to as the “long” protocol, to suppress endogenous gonadotropin secretion in the pre-stimulation phase to prevent a premature LH surge during the stimulation phase. Because of the decreased endogenous gonadotropins in the follicular phase, this protocol allows for a more synchronous cohort of follicles to grow. Disadvantages of this method include the longer duration of treatment and the possibility of a blunted response to gonadotropin therapy – which may increase the dose and duration of total gonadotropin administration.

Human chorionic gonadotropin (hCG) has similar activity to LH, given its close molecular structure (similar alpha peptide chain, but differing beta chain) and its ability to activate LH receptors on the ovary. During early pregnancy, hCG continues to induce progesterone production from the corpus luteum to maintain pregnancy viability (6). It has been used in ART cycles to trigger oocyte maturation and release since the 1970s (7).

A systematic review and meta-analysis of evaluating GnRH agonist versus GnRH antagonists in oocyte donation IVF treatment cycles showed no significant difference in ongoing pregnancy rate or number of retrieved oocytes after donor stimulation with GnRH agonist or antagonist protocols (9). A meta-analysis assessing oocytes retrieved as an outcome showed no difference in the number retrieved with GnRH agonist or antagonist use (10). However, a randomized controlled trial evaluating follicular growth and oocyte maturation resulted in a lower mean number of Metaphase II oocytes retrieved in the GnRH-antagonist group than the GnRH agonist group (11). The incidence of ovarian hyperstimulation syndrome (OHSS) was not seen to be significantly different between the two treatment groups based on a meta-analysis (10); however, there is mixed data on the subject.

## Ovulation trigger: Gonadotropin releasing hormone agonist versus human chorionic gonadotropin

The development of controlled ovarian hyperstimulation (COH) protocols using GnRH antagonists to suppress endogenous gonadotropin secretion permitted a GnRH agonist to be used as a trigger for endogenous luteinizing hormone release, and subsequent oocyte maturation and retrieval, instead of hCG (12–15). GnRH antagonists reversibly bind to the pituitary gland. When a bolus of GnRH agonist is given, the GnRH antagonist is displaced from the pituitary receptors by the GnRH agonist, activating the receptor, causing FSH and LH release. This LH surge lasts for a total of 24-36 hours, while the LH surge induced by hCG lasts for 48hrs, as LH has a half-life of about 60 minutes versus that of hCG which can last greater than 24 hours (16). Contrary to hCG, GnRH agonist stimulates both endogenous LH and FSH surges (7, 8). This mechanism more closely resembles the natural menstrual cycle.

The major benefit of GnRH agonist trigger over standard hCG trigger is the significant decrease in incidence of Ovarian Hyperstimulation Syndrome (OHSS), as outlined in numerous studies (17, 18), with one meta-analysis finding an odds ratio of 0.15 (95% confidence interval (CI) 0.05 to 0.47) for developing mild, moderate, or severe OHSS (12). hCG levels have been found to be positively associated with OHSS severity. Given the longer luteotropic action of hCG versus LH, there is suspected prolonged stimulation of LH receptors on corpora lutea with increased vascular endothelial growth factor (VEGF) release following hCG trigger use (19). For patients with a pronounced response to ovarian stimulation, GnRH agonist use instead of hCG as an ovulation trigger has been recommended to decrease the risk of OHSS and lead to a faster improvement in post-retrieval symptoms (7, 9, 20).

While the specific mechanisms leading to OHSS are unknown, the major cause is thought to be due to an LH-induced increase in VEGF release from the corpus luteum and release of other vasoactive peptides from granulosa cells (10). This leads to increased arteriolar vasodilation and vascular permeability, with fluid shifts from intra- to extravascular spaces (7, 19). OHSS usually arises about 4 to 14 days following ovarian stimulation, with presenting symptoms of abdominal distention, pain, edema, ascites, enlarged ovaries and cysts. Complications include jaundice, liver test abnormalities, hemoconcentration, electrolyte imbalances, and hypercoagulability. In severe cases, acute renal insufficiency, venous thromboembolism, and pleural effusions can result (19). Though rare, death can result from sepsis, dehydration and shock (6). It is estimated that approximately 20-33% of ART cycles are affected by mild OHSS, and 1-8 percent of ART cycles by moderate-to-severe OHSS (11, 19).

This decreased risk of OHSS in patients undergoing a GnRH agonist trigger protocol is also seen in patients that are at high-risk for OHSS. Risk factors for OHSS include younger age (<35 years old), ovulation disorders such as polycystic ovarian syndrome (PCOS), unexplained infertility, high serum antimullerian hormone (>3.4 ng/mL) and peak estradiol levels (>3,500 pg/mL), high antral follicle count (>24), increased number off oocytes retrieved (>24), and possibly lower body mass index (19). A study comparing high-risk patients who received GnRH agonist trigger after cotreatment with a GnRH antagonist with luteal phase estrogen and progesterone support found no significant difference in the number of oocytes collected (20.2 vs 18.8), proportion of Metaphase II oocytes (81% vs 83.8%), fertilization rates (71.6% vs 74.9%), implantation rates (36% vs 31%), clinical pregnancy rate (56.7% vs 51.7%), and ongoing pregnancy rate (53.3% vs 48.3%) in GnRH agonist vs hCG trigger groups. However, midluteal ovarian volume was significantly increased the in the hCG trigger group (36.6 cm^3^ vs 129 cm^3^, p<0.01), and the percentage of patients with OHSS was significantly increased in the hCG trigger group (0% vs 31.3%, p<0.01) (17).

Additional benefits of GnRH agonist trigger instead of hCG are increased oocyte maturity, increased proportion of oocytes resuming meiosis and increased number of Metaphase II oocytes (7). Humaidan et al. showed that patients who received a GnRH agonist trigger had a significantly greater proportion of Metaphase II oocytes (84% versus 68%, p<0.02) in ICSI exposed oocytes than those who received a hCG trigger. Rates of fertilization were similar (60% versus 54%) between groups (21). Kolibianakis et al. also found a similar proportion of Metaphase II oocytes (73% vs 78%), fertilization rates (55% vs 58%), and number of two-pronuclei (2PN) oocytes (5.1 vs 5.8) following GnRH agonist or hCG trigger, respectively (22). This increased oocyte maturity is thought to be secondary to the endogenous FSH surge induced by GnRH agonists (23), which is not induced by hCG. Even in patients who are classified as normal and low responders, GnRH agonist trigger use has been shown to be effective in obtaining mature oocytes. A study by Maslow et al. found similar oocyte maturity rates regardless of peak estradiol level following GnRH agonist trigger, though they noted that total number of oocytes declined with decreasing estradiol levels (9). Studies have also shown that the addition of a GnRH agonist to an hCG trigger (dual trigger approach) leads to increased fertilization rates in IVF compared to an isolated hCG trigger (24). A meta-analysis was performed comparing this dual trigger to hCG alone and found an increased number of mature oocytes collected, increased number of fertilized oocytes, increased implantation, clinical pregnancy, and live birth rates in the dual trigger group. Importantly, an increased risk of OHSS was not associated with dual trigger use (8).

The major drawback to GnRH agonist trigger use has been decreased rates of clinical pregnancy, embryo implantation, and live birth rates in fresh embryo transfer cycles (12, 25), and increased miscarriage rates (21, 22). The ongoing hypothesis for this finding of decreased clinical pregnancy rates is due to decreased corpus luteum function and endometrial receptivity, resulting from the decreased luteinizing hormone (LH) surge induced by a GnRH agonist trigger versus hCG. These disadvantages are important to consider; however, more data is needed to make conclusions for frozen autologous cycles.

## Fertility preservation

GnRH agonist trigger protocols have been recommended over an hCG trigger in oocyte donors and cancer patients undergoing fertility preservation. This recommendation is based off the assumption that these patients tend to be young women with sufficient ovarian reserve, who respond well to ovarian stimulation; therefore, they are at an increased risk of OHSS than patients with low response to ovarian stimulation (7). Additionally, as they will not be undergoing an immediate embryo transfer, optimal endometrial conditions are not required, so a prolonged luteal phase is not needed. A study in cancer patients undergoing fertility preservation found that patients triggered with a GnRH agonist had increased number and percentage of oocytes in the Meiosis II phase, as well as increased fertilization rates and increased number of 2PN embryos compared to patients treated with hCG trigger (23). This coincides with other authors’ recommendations for GnRH agonist trigger use in freeze-all cycles and oocyte donation programs to minimize OHSS risk without negatively impacting pregnancy outcomes (11, 26).

Of note, the above-mentioned findings of decreased ongoing pregnancy rates and birth rates along with increased miscarriage rates in patients receiving a GnRH agonist trigger versus hCG trigger were seen in fresh autologous cycles. This is not an issue in patients undergoing fertility preservation, as all oocytes or created embryos will be frozen. Importantly, oocytes and embryos derived from COH protocols using a GnRH that are then frozen and later fertilized and/or transferred have similar pregnancy and live birth rates when compared to oocytes/embryos derived for COH protocols using a hCG trigger. In the Cochrane review by Youseff, et al., patients undergoing donor-recipient cycles had similar clinical pregnancy rates (OR 0.87, 95% CI 0.57 to 1.33) and live birth rates compared to those receiving hCG trigger (OR 0.92, CI 0.53-1.61), but a significantly decreased incidence of OHSS (OR 0.05, 95% CI 0.01 to 0.28) (12). Two other studies showed similar findings in oocyte donors. Donors who received a GnRH agonist trigger versus hCG trigger had similar number of retrieved oocytes, percentage of METAPHASE II oocytes (70% vs 76%), similar rates of fertilization (80% vs 65%), implantation (29% vs 32%), and pregnancy (55% vs 59%), but a significantly decreased rate of OHSS (0% vs 16.6%) (27). Galindo et al. found no significant differences in ongoing pregnancy and live birth rates, but there were no cases of OHSS in the oocyte donors treated with GnRH agonist trigger but 9% of donors treated with hCG trigger developed OHSS (28).

However, it should be noted that use of GnRH agonist trigger does not completely eliminate the risk for OHSS. Two case reports described development of severe OHSS, characterized by abdominal ascites requiring drainage and hemoconcentration, in patients treated with GnRH agonist trigger in a GnRH antagonist protocol. One patient had polycystic appearing ovaries on baseline ultrasound prior to ovarian stimulation, while the second patient had no prior medical problems and history of regular menstruation (29). Regardless, in a Delphi Consensus comprising of global experts in the ART field, 100% of experts surveys agreed that a GnRH agonist trigger, in a GnRH antagonist protocol, should be used for final oocyte maturation in women at risk of OHSS (30). This further supports the use of GnRH agonist trigger protocols for patients undergoing fertility preservation, regardless of reason.

Fertility preservation is not only an important consideration for patients with genetic conditions but also those with breast cancer patients, given 25% of breast cancers occur in young patients prior to entering menopause (31). GnRH agonist trigger has been shown to be effective for fertility preservation in breast cancer patients. Oktay et al. found that in breast cancer patients undergoing a Controlled Ovarian Stimulation Treatment with Letrozole Supplementation Study protocol [COST-LESS (32)], patients treated with a GnRH agonist trigger had a similar number of total oocytes retrieved as those treated with a hCG trigger (16.4 ± 10.3 versus 12.8 ± 7.7, NS). However, a GnRH agonist trigger showed benefit in terms of significantly increase number of metaphase II (METAPHASE II) oocytes (11.9 ± 6.6 versus 7.4 ± 4.9, p<0.001), increased mean maturation rate (77.3 ± 21.1% vs 68.5 ± 23.3%, p=0.049), and increased mean fertilization rate (84.1 ± 11.1% vs 74.0 ± 24.9%, p=0.027) with an increased number of 2PN embryos (9.3 ± 5.7 vs 6.3 ± 4.6, p=0.008), and significant decrease in development of mild/moderate OHSS (3.7% vs 21.3%, p=0.047). An additional benefit of GnRH agonist trigger use was the significant drop in oestradiol concentrations from trigger day to 4 days post-trigger (89.5% ± 6.3% vs 79.0 ± 13.4%, p=0.013) (33). There have been concerns for cancer progression in patients with estrogen-responsive breast cancer undergoing ovarian stimulation and oocyte/embryo cryopreservation, given the increased oestrogen exposure that occurs during these treatments. Use of the COST-LESS protocol causes lower peak oestradiol concentrations during controlled ovarian stimulation with no change in number of embryos obtained or fertilization rates (34), and without increasing risk of breast cancer recurrence (35). Use of a GnRH agonist trigger instead of an hCG trigger is proposed to make fertility preservation safer for breast cancer patients by further decreasing oestrogen exposure. This benefit in addition to the obtainment of mature oocytes with improved fertilization rates, has led to its recommendation as the primary trigger in oestrogen-sensitive breast cancer patients (33).

An additional benefit of GnRH agonist trigger for fertility preservation in patients with malignancy is its success in a Random Start Controlled Ovarian Stimulation protocol. This protocol involves the use of letrozole and gonadotropins with GnRH antagonist for COH, beginning at any point during the menstrual cycle. Sonmezer et al. first showed successful oocyte retrieval and embryo creation with mean maturity and fertilization rates of 58.8-77.7% and 69.2%- 87.5%, respectively, in breast cancer patients undergoing a Random Start protocol, with COH starting on menstrual days 11, 14, or 17 (36). This study used a hCG trigger. Ozkaya et al. reported successful use of a GnRH agonist trigger in a breast cancer patient undergoing the Random Start protocol. Thirty-one oocytes were retrieved, 52% were mature, and 22 oocytes were vitrified. The patient also did not develop OHSS and began chemotherapy 48hr after oocyte retrieval (37).

## Conclusion

Fertility Preservation in patients with inherited genetic conditions requiring HSCT is a developing field. As these treatments often cause infertility, counseling patients about their options for fertility preservation prior to treatment is of utmost importance. If patients elect for oocyte cryopreservation, ART protocols utilizing GnRH agonist triggers offers the greatest chance of obtaining high quality, mature oocytes with the more favorable side effect profile, especially decreased rates OHSS, which could otherwise delay treatment.

## Author contributions

JM, CM and AD had substantial contributions to the gathering and compilation of data, in addition to the formulation of the manuscript. All authors contributed to the article and approved the submitted version.

## Conflict of interest

The authors declare that the research was conducted in the absence of any commercial or financial relationships that could be construed as a potential conflict of interest.

## Publisher’s note

All claims expressed in this article are solely those of the authors and do not necessarily represent those of their affiliated organizations, or those of the publisher, the editors and the reviewers. Any product that may be evaluated in this article, or claim that may be made by its manufacturer, is not guaranteed or endorsed by the publisher.
